# Exposure to Molybdate Results in Metabolic Disorder: An Integrated Study of the Urine Elementome and Serum Metabolome in Mice

**DOI:** 10.3390/toxics12040288

**Published:** 2024-04-14

**Authors:** Kun Zhou, Miaomiao Tang, Wei Zhang, Yanling Chen, Yusheng Guan, Rui Huang, Jiawei Duan, Zibo Liu, Xiaoming Ji, Yingtong Jiang, Yanhui Hu, Xiaoling Zhang, Jingjing Zhou, Minjian Chen

**Affiliations:** 1 State Key Laboratory of Reproductive Medicine and Offspring Health, Center for Global Health, School of Public Health, Nanjing Medical University, Nanjing 211166, China; zk@njmu.edu.cn (K.Z.); tmm_2023@163.com (M.T.); cyanling9211@163.com (Y.C.); guanys000@163.com (Y.G.); hrye0928@163.com (R.H.); jwduan1997@163.com (J.D.); liuzibo@njmu.edu.cn (Z.L.); jxmnjmu@163.com (X.J.); ytjiang61@163.com (Y.J.); zhou_jingjing56@163.com (J.Z.); 2 Key Laboratory of Modern Toxicology of Ministry of Education, School of Public Health, Nanjing Medical University, Nanjing 211166, China; 3 Department of Epidemiology, Center for Global Health, School of Public Health, Nanjing Medical University, Nanjing 211166, China; 4 Sir Run Run Hospital of Nanjing Medical University, Nanjing 211166, China; wzhang_nj@foxmail.com (W.Z.); njjshyh@126.com (Y.H.); 5 Department of Hygienic Analysis and Detection, Nanjing Medical University, Nanjing 211166, China; zhangxl3@njmu.edu.cn

**Keywords:** molybdate, cadmium, elementome, metabolomics, toxicity

## Abstract

The increasing use of molybdate has raised concerns about its potential toxicity in humans. However, the potential toxicity of molybdate under the current level of human exposure remains largely unknown. Endogenous metabolic alterations that are caused in humans by environmental exposure to pollutants are associated with the occurrence and progression of many diseases. This study exposed eight-week-old male C57 mice to sodium molybdate at doses relevant to humans (0.01 and 1 mg/kg/day) for eight weeks. Inductively coupled plasma mass spectrometry (ICP-MS) and ultra-performance liquid chromatography tandem mass spectrometry (UPLC-MS) were utilized to assess changes in urine element levels and serum metabolites in mice, respectively. A total of 838 subjects from the NHANES 2017–2018 population database were also included in our study to verify the associations between molybdenum and cadmium found in mice. Analysis of the metabolome in mice revealed that four metabolites in blood serum exhibited significant changes, including 5-aminolevulinic acid, glycolic acid, l-acetylcarnitine, and 2,3-dihydroxypropyl octanoate. Analysis of the elementome revealed a significant increase in urine levels of cadmium after molybdate exposure in mice. Notably, molybdenum also showed a positive correlation with cadmium in humans from the NHANES database. Further analysis identified a positive correlation between cadmium and 2,3-dihydroxypropyl octanoate in mice. In conclusion, these findings suggest that molybdate exposure disrupted amino acid and lipid metabolism, which may be partially mediated by molybdate-altered cadmium levels. The integration of elementome and metabolome data provides sensitive information on molybdate-induced metabolic disorders and associated toxicities at levels relevant to human exposure.

## 1. Introduction

Molybdenum is an essential trace element for microorganisms, plants, and animals, playing a crucial role in maintaining metabolic homeostasis [1]. In the form of molybdate, the element molybdenum finds extensive applications in food, industry, and medicine. For instance, it is utilized in the production of promising nanomaterials and layered structural materials [2,3]. Common routes of molybdenum exposure in the general population include air, soil, water, and food [4]. The absorption rate of molybdenum in the human body depends on the solubility of the various forms of molybdenum, such as molybdenum disulfide (MoS_2_, insoluble in water) and sodium molybdate (water solubility: 840 g/L in water at 100 °C) [5]. Molybdenum is primarily absorbed from the gastrointestinal tract in the form of the molybdate anion, which subsequently binds with albumin and is predominantly excreted through urine [6]. For the percentage of molybdate absorbed from the gastrointestinal tract, it has been reported that molybdenum was very efficiently absorbed (88–93%), at all dietary molybdenum intakes [6]. The widespread use of molybdenum can only cause increased human exposure if there is an exposure pathway (e.g., inhalation of molybdenum containing dust) [7]. Molybdenum is an essential element, excessive exposure to molybdate has been associated with adverse health outcomes [8]. The literature reports have indicated the harmful effects of high-dose molybdate exposure (10–50 mg/kg) in animal models [9,10]. Considering the narrow safety range of essential trace elements, it is crucial to investigate the potential toxic effects of molybdate at human exposure levels. However, our current understanding of these effects remains significantly limited.

Excessive molybdate exposure can trigger changes in endogenous metabolism in the human body, which may contribute to the development of various diseases such as obesity, diabetes, cardiovascular diseases, reproductive abnormalities, and cancer [11]. For instance, lipid metabolism disorders are linked to cardiovascular diseases, while oxidative stress is associated with organ damage. Therefore, investigating the metabolic disorders caused by molybdate exposure is crucial, as it can indicate the potential toxicity of molybdate. In recent years, metabolomics has emerged as a complementary technology to genomics, transcriptomics, and proteomics, focusing on revealing gene expression outcomes [12,13]. Metabolomics, being closer to the organism’s phenotype, enables the simultaneous observation of changes in numerous metabolites [14]. As metabolomics can reflect the physiological or pathological state of organisms, it plays a vital role in studying the effects and underlying mechanisms of environmental chemical toxicity [15]. Liquid chromatography coupled to tandem mass spectrometry (LC-MS) is a powerful tool for identifying and classifying metabolomes for its high sensitivity and wide range of chemical detection coverage [16]. Therefore, it is necessary to employ metabolomics technology to investigate the effects of molybdate exposure on the body’s endogenous metabolism.

One of the major mechanisms underlying the toxicity of metals is their interaction with other elements in the body [17]. However, the effect of molybdate exposure on other elements is still largely unknown [18,19]. Importantly, altered exposure to elements can lead to metabolic changes in the body. For example, long-term exposure to copper can interfere with lipid metabolism [20]. Iron overload induces free radical formation, lipid peroxidation, DNA and protein damage, leading to carcinogenesis or ferroptosis [21]. Magnesium, as an essential cofactor, actively participates in carbohydrate metabolism and regulates energy metabolism and blood sugar control [22]. Therefore, the metabolic disorders resulting from molybdate exposure may be caused by disruptions in element levels in the body. Elementomics is an emerging omics technology that aims to analyze dozens of elements simultaneously, providing a comprehensive understanding of changes in their concentration in various body fluids. Hence, it is necessary to investigate the detailed effects of molybdate exposure on different elements using elementomics analysis.

Urine is a commonly used matrix for studying the body’s exposure burden, while blood samples can be used to explore general metabolic changes in the body. The novel integration of elementomics and metabolomics can provide information on the disruption of the metabolome through the elementome, which has been rarely reported in previous studies.

This study utilized an animal model to investigate the effects of molybdate exposure at levels relevant to human exposure. It examined the influence of molybdate exposure on the elementome and metabolome, revealing a potential association between molybdate exposure and metabolic disorders. Meanwhile, the major findings between molybdate exposure and elementome were also verified in humans. These findings provide a novel perspective on the potential toxicity of molybdate and contribute to our understanding of this issue.

## 2. Materials and Methods

### 2.1. Experimental Materials

Sodium molybdate (purity ≥ 99%) was purchased from Rhawn Reagent [Shanghai, China, https://www.rhawn.cn (accessed on 21 December 2023)]. Methanol (purity ≥ 99%) (Merck, Darmstadt, Germany) and acetonitrile (purity ≥ 99%) (Merck, Darmstadt, Germany) were utilized in this study. The standard compounds were purchased from Sigma-Aldrich (St. Louis, MO, USA), Adamas Reagent Co., Ltd. (Shanghai, China), and Aladdin Reagent Company (Shanghai, China). Deionized water (resistivity ≥ 18.2 M Ω cm) was obtained using a Milli-Q system (Millipore, Milford, MA, USA). Nitric acid (65–70%, *w*/*w*, ≥99.9999%, trace metals basis) was purchased from Alfa Aesar Ltd. (Tianjin, China). The Multielementary solutions including IV-ICPMS-71A (10 ppm 43 Element (Al, As, Ba, Be, Cd, Ca, Ce, Cr, Co, Cu, Dy, Er, Eu, Gd, Ga, Ho, Fe, La, Pb, Lu, Mg, Mn, Nd, Ni, P, K, Pr, Rb, Sm, Se, Ag, Na, Sr, S, Tl, Th, Tm, U, V, Yb, Zn, Cs, and B), 3% *v*/*v* Nitric Acid), IV-ICPMS-71B (10 ppm Refractory Element (Sb, Ge, Hf, Mo, Nb, Si, Ta, Te, Sn, Ti, W, and Zr), 3% *v*/*v* Nitric Acid/trace Hydrofluoric Acid), IV-ICPMS-71C (10 ppm Precious Metal (Au, Os, Pt, Rh, Ir, Pd, Re, and Ru), 30% *v*/*v* Hydrochloric Acid), CCS-4 (100 ppm Aklali, Alkaline Earth, Non-Transition Elements (Al, As, Ba, Be, Bi, Ca, Cs, Ga, In, Li, Mg, K, Rb, Se, Na, and Sr), 7% *v*/*v* Nitric Acid), AAHG1 (1000 μg/mL mercury, 5% *v*/*v* Nitric Acid), MSAU (100 μg/mL gold HCl, 10% *v*/*v* Hydrochloric Acid), MSLI (100 μg/mL lithium, 0.1% *v*/*v* Nitric Acid), and an internal standard IV-ICPMS-71D (10 ppm 6 Element (Bi, In, Sc, Tb, Y, and ^6^Li), 3% *v*/*v* Nitric Acid) were purchased from Inorganic Ventures (Christiansburg, VA, USA). The elements in the solutions can be found on the website [https://www.inorganicventures.com (accessed on 21 December 2023)].

### 2.2. Animal Experiment

Eight-week-old male C57 mice, bred under specific pathogen-free (SPF) conditions, were obtained from the Laboratory Animal Center of Nanjing Medical University in Nanjing, China. To avoid the impact of estrous cycle on metabolism in female mice, only male mice were selected. After one week of adaptive feeding, the mice were randomly divided into control and treatment groups. The administration was conducted via gavage. The control, low-exposure, and high-exposure groups were given sodium molybdate at doses of 0, 0.01, and 1 mg/kg/day, respectively, corresponding to human exposure levels. The selection of the low dose of 0.01 mg/kg/day was based on its ability to generate a toxicity burden similar to tolerable upper intake level (UL) and minimal risk level (MRL) in humans. The European Commission’s Scientific Committee on Food has set the UL of molybdenum at 0.6 mg/day for adults, which is equivalent to 0.01 mg/kg/day considering an average weight of 60 kg [23]. This dose is also close to the previously reported MRL of 0.008 mg/kg/day [24]. The dose of 1 mg/kg/day was selected based on the range of human occupational exposure [25]. The exposure period lasted for 8 weeks. To improve statistical power, the ratio of the number of mice in the treatment group to the control group was set at 1:1.5. Based on animal welfare considerations and sample size estimation using 3Rs-Reduction.co.uk, 5 mice were used in the control group, and 3 mice were used in each of the treatment groups. This sample size was determined based on a signal-to-noise ratio of the toxic effects including metabolite and the element changes ranging from 2.0 to 2.8 observed in our pilot study. All mice had ad libitum access to food and water and were housed in a controlled and standardized laboratory environment with a temperature ranging from 20 to 26 °C, relative humidity ranging from 40 to 70%, and a 12 h light/dark cycle. The body weights of the mice were recorded weekly. Fasting urine and blood samples were collected, and the mice were then sacrificed. The organs, including the heart, lung, liver, spleen, kidney, and intestine, were weighed. This study strictly adhered to international standards on animal welfare and the guidelines of the Institute for Laboratory Animal Research of Nanjing Medical University. All procedures conducted in this study were approved by the Animal Ethical and Welfare Committee of Nanjing Medical University (IACUC-2008055).

### 2.3. Histological Examination

Heart, liver, spleen, lung, kidney, and intestine samples were collected, fixed in 4% paraformaldehyde and then embedded in paraffin [26]. Afterwards, the tissues were cut into sections (5 μm), which were deparaffinized, rehydrated, and stained with hematoxylin (0.1%) for 10 min, and eosin (0.1%) for 5 min. Hematoxylin and eosin (HE) stained sections were digitalized with the whole-slide Pannoramic MIDI scanner (3DHISTECH Ltd., Budapest, Hungary) at 20× magnification and analyzed with Pannoramic Viewer software (3DHISTECH, Budapest, Hungary).

### 2.4. Analysis of Elementome in Urine

The detection of the elementome in urine using the standard curve method for quantification was conducted following our previous reports [27]. Prior to the experiment, all glassware was soaked in 10% nitric acid for 24 h, rinsed with deionized water 8 to 10 times, and dried in a vacuum drying cabinet at 37 °C for later use. For each sample, 10 μL of urine was added to 485 μL of 1% dilute nitric acid, and 5 μL of internal standard IV-ICPMS-71D (10 ppm 6 Element (Bi, In, Sc, Tb, Y, and ^6^Li), 3% *v*/*v* Nitric Acid) (Inorganic Ventures, Christiansburg, VA, USA) at the concentration of 1 mg/L was added. The mixture was thoroughly mixed. The samples were then quantified using an iCAP Qc inductively coupled plasma mass spectrometry (ICP-MS) instrument (Thermo Fisher Scientific, Bremen, Germany). The Multielementary solutions (Inorganic Ventures, Christiansburg, VA, USA) were used in elementome analysis. The employed ICP-MS was equipped with a collision cell, and helium (99.999% grade) at 5.0 mL/min was used for the collision cell to remove polyatomic interferences. Quality control samples and blank samples were analyzed in parallel with the study samples. The limit of detection (LOD) was calculated as 3 times the standard deviation for 10 consecutive blank samples [28]. The concentration of undetectable urinary elements was imputed with LOD/2 according to the previous report [29]. The recoveries of the detected elements were between 86.8% and 107%.

### 2.5. Analysis of Metabolome in Serum

The blood serum metabolomics detection was conducted following our previously reported method [30]. Blood was collected by retro-orbital bleed into 1.5 mL Eppendorf tubes directly and allowed to clot for 30 min, followed by centrifugation at 3000× *g* for 10 minutes at 4 °C to collect serum (200–300 μL). Then, protein precipitation of blood serum (20 μL) was performed using methanol at a volume ratio of 1:3. After centrifugation at 20,000× *g* for 15 min at 4 °C, the supernatant was transferred. The target analytes were dried in a vacuum concentrator (Labconco, MO, USA) at room temperature and reconstituted with 20 μL deionized water for further analysis. Ultra-high performance liquid chromatography (UPLC) (Dionex, Germering, Germany) equipped with a Hypersil GOLD C18 column (100 mm × 2.1 mm, 1.9 μm, column temperature at 40 °C) and tandem Q Exactive Orbitrap and triple quadrupole mass spectrometry (Thermo Fisher Scientific, Bremen, Germany) were used in the analysis. The full scan mode was employed, ranging from 70 *m*/*z* to 1050 *m*/*z*, at a resolution of 70,000 with the heated electrospray ionization (HESI) source. For UPLC analysis, a multistep gradient was used with mobile phase A consisting of 0.1% formic acid in water and mobile phase B consisting of 0.1% formic acid in acetonitrile. The flow rate was set at 0.4 mL/min over a run time of 17 min. Gradient program was conducted as follows: 0–3 min, 1% mobile phase B and 99% mobile phase A; 3–10 min, 1–99% mobile phase B and 99–1% mobile phase A; 10–15 min, 99% mobile phase B and 1% mobile phase A; 15–17 min, 1% mobile phase B and 99% mobile phase A. The autosampler temperature was maintained at 4 °C, and the injection volume was 10 μL. The mass spectrometer parameters were set as follows: for the positive mode, a spray voltage of 3.5 kV; for the negative mode, a spray voltage of 2.5 kV; for both modes, a capillary temperature of 300 °C; a sheath gas flow of 50 arbitrary units (AU); an auxiliary gas flow of 13 AU; a sweep gas flow of 0 AU; and an S-Lens RF level of 60. Metabolite identification was based on the comparison of accurate mass and retention time with authentic metabolite standards. The analysis was conducted in a randomized fashion to avoid complications related to the injection order. An equivalent volume from each serum sample was mixed to create quality control (QC) samples. The same procedural steps applied to the test samples were followed for the QC samples, which were injected after every fifth sample injection during the analysis.

### 2.6. Human NHANES Population Study

**Figure 1:**
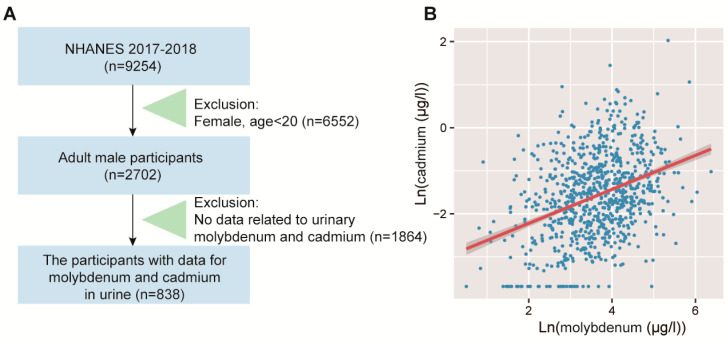
Correlation analysis of urinary molybdenum and cadmium in the NHANES population. (**A**) Participant screening process for NHANES 2017–2018. (**B**) Scatter plot illustrating the linear regression between molybdenum and cadmium. Data of molybdenum and cadmium were ln-transformed before analysis.

We conducted an analysis using NHANES data from 2017 to 2018, which included years with available urine metal exposure data. The participant selection process is illustrated in Figure 1A. Specifically, to avoid the impact of menstrual cycles on metabolism, we included only adult male subjects who had complete data for total molybdenum and cadmium in urine. Ultimately, a total of 838 subjects were included in our study. All data can be downloaded from the official website [https://www.cdc.gov/nchs/nhanes (accessed on 21 December 2023)]. The Centers for Disease Control and Prevention (CDC) Research Ethics Review Board approved the project, and all participants gave informed consent.

### 2.7. Assessment of Molybdenum and Cadmium Exposure in NHANES

Spot urine samples were analyzed for molybdenum and cadmium using inductively coupled plasma-dynamic reaction mass spectrometry (ICP-DRC-MS) at the National Center for Environmental Health, CDC. For values below the LOD, imputation was performed using the LOD value divided by the square root of two for each metal. The laboratory analyses followed the protocols described in the previous study [31].

### 2.8. Statistical Analysis

**Figure 2:**
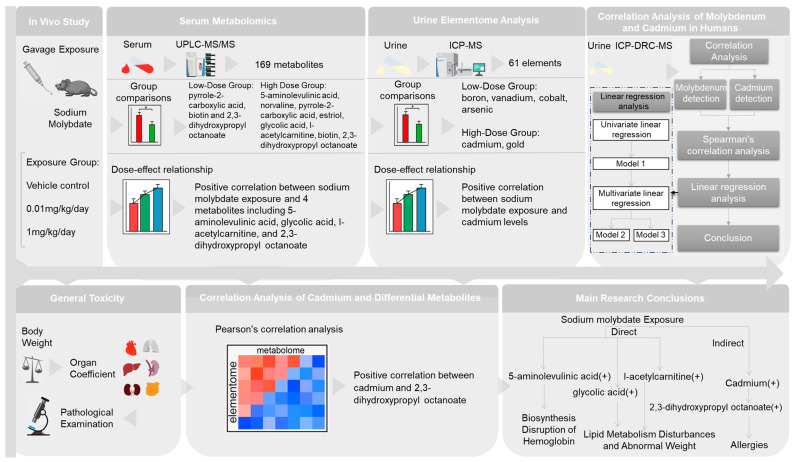
Overview of the study design and proposed effects of molybdate exposure on metabolic disorders associated toxicities, either directly or indirectly, through disruption of element. “+” indicates a positive correlation; * *p* < 0.05. The control group is represented by red column, the 0.01 mg/kg/day group by green column, and the 1 mg/kg/day group by blue column in the dose-effect relationship analysis.

For the animal study, a *t*-test was used to analyze the differences between the two groups. When the comparison involved three groups, one-way analysis of variance (ANOVA) followed by Dunnett’s test was employed. The normality and homogeneity of variance for all data were assessed using the Kolmogorov–Smirnov test. To improve the robustness of the differential metabolites based on the dose–effect relationship, Spearman’s correlation analysis was conducted to investigate associations between molybdate and elementome, and molybdate and metabolome in the animal study. Pearson’s correlation test was used to explore the associations between differential elements and differential metabolites. In the population study using NHANES data, Spearman’s correlation analysis, and univariate and multivariate linear regression models were applied to study the association between molybdenum and cadmium. To account for potential confounding variables, age, race, education, smoking status, and BMI were included for adjustments in multivariate linear regression. Age was included as a continuous variable, and race, education, smoking status, and BMI were included as categorical variables. Model 1 was unadjusted; model 2 was controlled for age and race; model 3 was controlled for age, race, education, smoking status and BMI. The statistical analysis was performed using R (Version 4.0.5). Partial Least Squares Discrimination Analysis (PLS-DA) was utilized for dimensionality reduction analysis of elementome and metabolome data by SIMCA version 14.1 (Umetrics, Umea, Sweden). The statistical significance threshold was set at *p* < 0.05. The visualization of the metabolite network was established using iPath [https://pathways.embl.de (accessed on 21 December 2023)]. The study design can be found in Figure 2.

## 3. Results

### 3.1. The Effect of Molybdate Exposure on Body Weight, Organ Coefficients, and Histopathological Examination in Mice

In this study, we investigated the effect of molybdate exposure on various parameters in mice, including body weight, organ coefficients (weight of the organ (g)/total body weight (g) × 100), and histopathological examinations. During the exposure period, no significant differences in body weight were observed among mice in different groups at each time point (Figure S1A). Molybdate exposure did not lead to significant differences in the organ coefficients of the heart, liver, spleen, lung, kidney, and intestine in mice (Figure S1B). In the pathological analysis of the heart, liver, spleen, lung, kidney, and intestine, no evident histopathological changes were observed (Figure S1C). Overall, these results suggest that molybdate exposure does not significantly affect body weight, organ coefficients, or induce histopathological changes in the examined organs of mice.

### 3.2. The Effect of Molybdate Exposure on the Serum Metabolome in Mice

**Figure 3:**
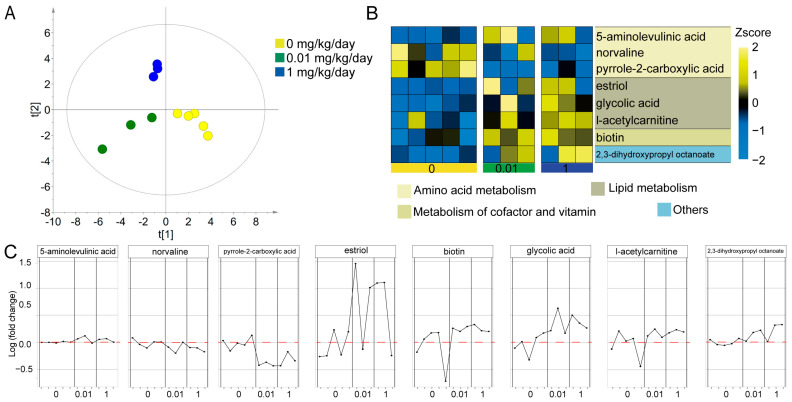
Overview of the effect of molybdate exposure on the serum metabolome. (**A**) PLS-DA score plots of metabolites, with each point representing a serum sample and colored according to the treatment groups. The control group is represented by yellow, the 0.01 mg/kg/day group by green, and the 1 mg/kg/day group by blue. The t [1] score on the *X*-axis represents the scores of the samples on the first latent variable (LV1) obtained from the PLS-DA model. The t [2] score on the *Y*-axis represents the scores of the samples on the second latent variable (LV2) obtained from the PLS-DA model. (**B**) Heatmap of differential metabolites. Yellow and blue colors represent increased and decreased levels of metabolites, respectively. (**C**) Changes in different metabolites in each sample of the different groups. A Z-score is a statistical measure that quantifies how many standard deviations a particular metabolite’s concentration is from the mean concentration of that metabolite across a set of samples. The Z-score is calculated using the formula: Z = (X − M)/SD. Z is the Z-score; X is the level of the metabolite; M is the mean level of the metabolite; and SD is the standard deviation of the level of the metabolite.

In this study, our hypothesis was that molybdate exposure could potentially influence metabolite profiles in mice. To explore this hypothesis, we conducted metabolomics analysis on serum samples. A total of 169 metabolites were quantified in mouse serum. The score plots of the PLS-DA model (Figure 3A) clearly demonstrated a distinct separation between the control and exposed groups (R^2^X = 0.649, R^2^Y = 0.993, Q^2^ = 0.430), indicating a significant impact of molybdate exposure on the metabolome profile in mice. To gain an overview of the detected metabolites, iPath was used to construct metabolic pathways (Figure S2). The metabolites were mainly enriched in amino acid metabolism, metabolism of cofactor and vitamin, and lipid metabolism (Figure 3B). Further analysis focused on the differential metabolites between the control and molybdate exposure groups. A total of eight different metabolites were identified (Figure 3B, C, Table 1). Pyrrole-2-carboxylic acid was decreased (*p* < 0.001) in the molybdate-0.01 mg/kg/day group compared to the control group, while biotin and 2,3-dihydroxypropyl octanoate were increased (*p* < 0.05). In the molybdate-1 mg/kg/day group, norvaline was slightly decreased (*p* < 0.05) and pyrrole-2-carboxylic acid was decreased (*p* < 0.05), while 5-aminolevulinic acid, estriol, glycolic acid, l-acetylcarnitine, biotin, and 2,3-dihydroxypropyl octanoate were increased (*p* < 0.05). Spearman’s correlation analysis between molybdate doses and metabolite levels revealed positive dose–effect correlations between molybdate and 5-aminolevulinic acid, glycolic acid, l-acetylcarnitine, and 2,3-dihydroxypropyl octanoate, respectively (r = 0.691, *p* < 0.05; r = 0.882, *p* < 0.05; r = 0.682, *p* < 0.05; r = 0.636, *p* < 0.05) (Table 1). These results significantly contribute to our understanding of the metabolic perturbations induced by molybdate exposure in mice, and identified 5-aminolevulinic acid, glycolic acid, l-acetylcarnitine, and 2,3-dihydroxypropyl octanoate as robust metabolic changes.

### 3.3. Molybdate Exposure Increased the Urinary Molybdenum Content and Affected the Urine Elementome in Mice

**Figure 4:**
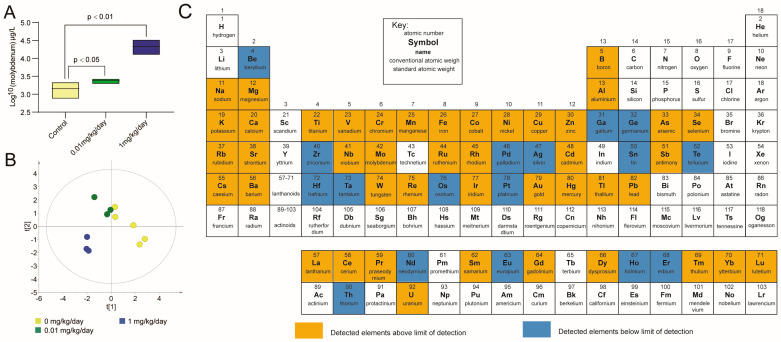
Overview of the effect of molybdate exposure on the urine elementome. (**A**) Box plots illustrating the total urinary molybdenum concentration in mice following molybdate treatment. (**B**) PLS-DA score plots of elements, with each point representing a urine sample and colored according to the treatment groups. The control group is represented by yellow, the 0.01 mg/kg/day group by green, and the 1 mg/kg/day group by blue. The *x*-axis and *y*-axis represent the scores obtained from the PLS-DA model. These scores are derived from the latent variables that capture the maximum covariance between the predictor variables (element levels) and the response variable (groups). The t [1] score on the *X*-axis represents the scores of the samples on the first latent variable (LV1) obtained from the PLS-DA model. The t [2] score on the *Y*-axis represents the scores of the samples on the second latent variable (LV2) obtained from the PLS-DA model. (**C**) Summary of the elements texted in urine.

In this study, we investigated the internal exposure level following exposure of mice to molybdate and its impact on the urinary elementome profile in mice using ICP-MS analysis. After exposure to molybdate, a significant increase in total molybdenum levels was observed in mice in the 0.01 mg/kg/day group (*p* < 0.05) and the 1 mg/kg/day group (*p* < 0.01), as depicted in Figure 4A. PLS-DA was performed on the elementome data obtained from the urine samples. The score plots of the PLS-DA model (Figure 4B) clearly demonstrated a distinct separation between the control and exposed mice (R^2^X = 0.429, R^2^Y = 0.718, Q^2^ = 0.436), indicating a significant influence of molybdate exposure on the elementome profile in mice. Elementome analysis conducted on the urine samples detected a total of 61 elements (Figure 4C). As shown in Table 2, the results revealed a significant increase in the levels of boron, vanadium, cobalt, and arsenic after exposure to 0.01 mg/kg/day of molybdate (*p* < 0.05). Additionally, cadmium levels were found to be increased, while the levels of gold were decreased in the group treated with 1 mg/kg/day of molybdate (*p* < 0.05). Spearman’s correlation analysis between molybdate doses and element levels showed a positive correlation between molybdate and cadmium (r = 0.786, *p* < 0.05) (Table 2). These findings significantly contribute to our understanding of the elementome response to molybdate exposure in mice, especially the effect of molybdate on the body’s handling of cadmium.

### 3.4. Validation of the Positive Correlation between Urinary Molybdenum and Cadmium in Humans

To validate the correlation between molybdenum and cadmium observed in mice, we conducted a population study using NHANES data from 2017 to 2018 to analyze the relationship between urinary molybdenum levels and cadmium content. The study included 838 eligible participants, and their basic characteristics are presented in Table S1. The average age of the participants was 52.14 (±17.81) years. Among male adults, non-Hispanic whites (34.4%) constituted the largest ethnic group. In terms of education level, 22.1% had completed high school or below, while 26.1% were high school graduates or held a GED equivalent. Approximately 28.0% had some college education or an associate’s degree, and 23.6% were college graduates or held higher degrees. Among adult males, 47.3% had never smoked, 32.2% reported past smoking but were not currently smoking, and 20.5% reported current smoking. A large proportion of individuals (38.2%) were classified as obese based on their BMI. The means (standard deviations (SDs)) of cadmium and molybdenum were 0.34 (0.46) and 53.40 (51.70) μg/L, respectively.

Spearman’s correlation analysis revealed a positive correlation between urinary molybdenum and cadmium (r = 0.32, *p* < 0.01). In the present study, univariate and multivariate linear regression analyses were performed, and the results of model 1, model 2, and model 3 are presented in Table 3. In model 1, a significant positive correlation between molybdenum and cadmium was observed (*β* = 0.39, 95% CI 0.32–0.46). To account for potential confounding variables, additional linear regression models were constructed. Model 2 was partially adjusted for age and race, while model 3 was fully adjusted for age, race, education, smoking status, and BMI. The results demonstrated a significant positive correlation between cadmium and molybdenum in both model 2 (*β* = 0.44, 95% CI 0.38–0.50) and model 3 (*β* = 0.47, 95% CI 0.41–0.52, Figure 1B). These findings further support the validated positive correlation between urinary molybdenum and cadmium in the population. Consequently, we conducted further investigations to explore the impact of molybdate exposure on key metabolisms through the perturbations in cadmium.

### 3.5. Correlation between Urinary Cadmium and Differential Serum Metabolites in Mice

It has been reported [32] that exposure to inorganic mercury can influence the elementome, leading to subsequent changes in the metabolome of organisms. Based on this, we proposed a hypothesis that molybdate exposure could potentially modify the metabolome of mice by altering their elemental composition in urine. Then, based on the elementome and metabolome results, we observed a dose-dependent change (*p* < 0.05) in cadmium, 5-aminolevulinic acid, glycolic acid, l-acetylcarnitine, and 2,3-dihydroxypropyl octanoate after molybdate exposure. Consequently, we specifically examined the correlation between cadmium and the above four differential metabolites by Pearson’s correlation analysis. As shown in Table 4, 2,3-dihydroxypropyl octanoate showed a positive correlation with cadmium (r = 0.782, *p* < 0.01), suggesting that the increase in 2,3-dihydroxypropyl octanoate induced by molybdate exposure might be mediated by cadmium (Figure 2).

## 4. Discussion

In this study, we did not observe any significant effects of molybdate exposure on body weight, organ coefficients, and histopathological examinations in mice. However, the analysis of the association between molybdate and the metabolome revealed four dose-related metabolite changes after exposure. These findings highlighted the sensitivity of omics technologies in detecting the effects of molybdate exposure and its potential toxicity, even at levels relevant to human exposure. Moreover, the integrated multi-omics analysis identified potential metabolic disturbances resulting from the altered levels of elements caused by molybdate exposure.

### 4.1. The Direct Effects of Molybdate Exposure on Serum Metabolome

Correlation analysis between molybdate exposure and the metabolome revealed that molybdate exposure mainly affects the levels of several metabolites involved in amino acid metabolism and lipid metabolism. Since these metabolic pathways are associated with various biochemical processes, it indicates that molybdate-induced metabolic disorders can lead to certain metabolic toxic effects. Molybdate can directly influence the levels of various metabolites, partially due to its role as a cofactor of flavoenzymes involved in diverse metabolic pathways. Exposure to molybdate can have significant effects on organisms by disrupting metabolism. In our study, we observed significant changes in four metabolites including 5-aminolevulinic acid, glycolic acid, l-acetylcarnitine, and 2,3-dihydroxypropyl octanoate following molybdate exposure in a dose-related manner.

5-aminolevulinic acid is an amino acid that plays a crucial role in the biosynthesis of heme, an essential component of hemoglobin [33]. Interestingly, when humans were exposed to molybdate at normal levels, there was a significant increase in the serum levels of 5-aminolevulinic acid, a key metabolite involved in the biosynthesis of hemoglobin [34]. Another study demonstrated that 5-aminolevulinic acid has the ability to affect the synthesis of porphyrin and hemoglobin, suggesting that 5-aminolevulinic acid may have a potential role in affecting erythropoiesis and changing hemoglobin production in certain conditions related to impaired red blood cell formation [35]. These findings provided evidence supporting previous reports that molybdenum, as a trace element, plays a crucial role in affecting the synthesis and function of hemoglobin [18].

Molybdate has the ability to form a complex with glycolic acid [36], which inhibits the normal excretion of glycolic acid, thereby explaining the increased levels of glycolic acid caused by molybdate. Glycolic acid, the smallest α-hydroxy acid, has effects on changes in human skin condition [37]. Simultaneously, glycolic acid can contribute to obesity by inhibiting the activity of lipase [38]. Glycolic acid has been used to distinguish between metabolically unhealthy obese participants and metabolically healthy obese subjects in a previous study [39]. L-acetylcarnitine (also known as acetyl-l-carnitine or ALCAR) is a compound derived from l-carnitine [40]. It has been studied for its potential effects on lipid metabolism and obesity [41]. Several studies have suggested that l-acetylcarnitine may play a potential role in weight changes through regulating fat oxidation and energy expenditure [42].

### 4.2. The Effects of Molybdate Exposure on Urine Elementome

Previous studies have demonstrated the potential of molybdate to influence the concentration of other elements within an organism [43]. Molybdenum functions as an active cofactor for molybdenum enzymes, playing a crucial role in various metabolic processes [44]. Furthermore, molybdate can influence the absorption and excretion of other elements, thereby leading to fluctuations in tissue element levels [45]. Notably, a positive correlation was found between molybdenum and cadmium in urine of mice and humans in this study. Cadmium is a non-essential element known for its toxicity. Once cadmium enters the body, it is challenging to eliminate completely, due to the absence of an efficient excretion mechanism, leading to potential accumulation [46]. It has been demonstrated that ducks fed with basal diet with different concentrations of molybdenum or/and cadmium influenced the concentration of trace elements in the digestive organs, in which a strongly positive correlation between molybdenum and cadmium was observed [47]. There are also other studies indicating a positive correlation between molybdenum and cadmium in humans [48]. Cadmium is a food-chain contaminant that has high rates of soil-to-plant transference, which makes dietary Cd intake unavoidable in both humans and animals. In this study, we randomly selected mice into different groups, used a high purity sodium molybdate standard (purity ≥ 99%), and no differences in food and water consumption among different mice groups were detected, confirming that the change of urinary cadmium was caused by molybdate exposure. In line with these findings, we also observed a positive correlation between molybdate and cadmium in mice, and this positive correlation was validated in the human population, suggesting that molybdate may impact the toxic element cadmium and exert toxic effects.

### 4.3. The Indirect Effects of Molybdate on Serum Metabolome through Urine Elementome

2,3-dihydroxypropyl octanoate, also known as caprylic acid triglyceride or octanoic acid triglyceride [49], is a triglyceride composed of three caprylic acid molecules esterified to a glycerol backbone [50]. It is a common lipid molecule in biological samples [51]. It has been reported that certain individuals may experience allergies with increased 2,3-dihydroxypropyl octanoate [52]. Interestingly, it is reported that alloys containing molybdenum may induce allergies such as eczema and impaired wound [53], suggesting that the up-regulation of 2,3-dihydroxypropyl octanoate caused by molybdate exposure in this study might promote allergies in mice. Notably, a study shed light on the potential role of cadmium in modulating allergic reactions and suggested a possible correlation between cadmium intake and allergies including ear swelling and edema in rats [54], suggesting that molybdate may affect 2,3-dihydroxypropyl octanoate through its association with cadmium and ultimately induce allergies.

## 5. Conclusions

In this study, a mouse model was used to investigate the effects of molybdate exposure, which is relevant to human exposure levels. By employing a novel approach that integrates elementome and metabolome data, a comprehensive understanding of the impact of molybdate exposure on body elements and metabolic profiles was obtained. While no significant change was observed at the given doses of molybdate in body weight, organ coefficients, and histopathological examinations, sensitive changes in the toxicity related metabolome were detected. Specifically, molybdate exposure disrupted amino acid and lipid metabolism in serum, which may be partially mediated by molybdate-altered cadmium levels. This study provides valuable insights into the potential toxicity and mechanisms of molybdate at levels relevant to human exposure. It also highlights the significance of integrating elementome and metabolome analyses in future toxicological research, particularly for studying metabolic disturbances and the underlying mechanisms related to elements.

## Figures and Tables

**Table 1:** Different metabolites in serum of mice caused by molybdate exposure.

Metabolite	0.01 mg/kg/day		1 mg/kg/day		0, 0.01, 1 mg/kg/day ^a^	
	Fold Change	*p*	Fold Change	*p*	r	*p*
5-aminolevulinic acid	1.145	0.100	1.100	0.041 *	0.691	0.019 *
norvaline	0.828	0.195	0.763	0.026 *	−0.573	0.066
pyrrole-2-carboxylic acid	0.397	0.000 *	0.504	0.011 *	−0.473	0.142
estriol	13.253	0.066	8.636	0.042 *	0.309	0.355
glycolic acid	2.458	0.056	2.422	0.008 *	0.882	0.000 *
l-acetylcarnitine	1.446	0.145	1.600	0.045 *	0.682	0.021 *
biotin	1.790	0.033 *	1.783	0.043 *	0.473	0.142
2,3-dihydroxypropyl octanoate	1.406	0.041 *	1.732	0.035 *	0.636	0.035 *

* *p* < 0.05. ^a^ Spearman correlation test using data from the 0, 0.01, 1 mg/kg/day groups.

**Table 2:** Different elements in urine of mice caused by molybdate exposure.

Element	0.01 mg/kg/day		1 mg/kg/day		0, 0.01, 1 mg/kg/day ^c^		LOD ^b^ (μg/L)
	Fold Change	*p*	Fold Change	*p*	r	*p*	
Boron	2.106	0.028 *	1.566	0.089	0.409	0.212	2.12
Vanadium	1.969	0.008 *	1.472	0.120	0.145	0.670	0.03
Cobalt	1.798	0.036 *	1.244	0.230	0.027	0.937	0.01
Arsenic	1.931	0.032 *	1.376	0.197	0.382	0.247	0.04
Cadmium	1.000	NA ^a^	25.578	0.000 *	0.786	0.004 *	0.08
Gold	0.533	0.296	0.192	0.037 *	−0.477	0.138	0.01

^a^ Cadmium was not detectable in the molybdate-0.01 mg/kg/day group. ^b^ The limit of detection. ^c^ Spearman correlation test using data from the 0, 0.01, 1 mg/kg/day groups. * *p* < 0.05.

**Table 3:** Multivariable associations of urinary molybdenum with cadmium.

Element	Model 1			Model 2			Model 3		
	No.	β (95%CI)	*p*	No.	β (95%CI)	*p*	No.	β (95%CI)	*p*
Cadmium	838	0.39 (0.32, 0.46)	*p* < 0.01	838	0.44 (0.38, 0.50)	*p* < 0.01	822	0.47 (0.41, 0.52)	*p* < 0.01

Model 1: unadjusted model. Model 2: adjusted for age and race. Model 3: adjusted for age, race, education, smoking status, and BMI.

**Table 4:** Correlation between urinary cadmium and differential serum metabolites.

Metabolite	Cadmium	
	r	*p*
5-aminolevulinic acid	0.025	0.942
glycolic acid	0.176	0.606
l-acetylcarnitine	0.413	0.207
2,3-dihydroxypropyl octanoate	0.782	0.004 *

* *p* < 0.05.

## Data Availability

Data are contained within the article.

## Supplementary Materials

The following supporting information can be downloaded at: https://www.mdpi.com/article/10.3390/toxics12040288/s1, Figure S1: General toxicity of molybdenum exposure in vivo; Figure S2: Detected metabolites in the general metabolic pathway based on iPath 3.0 [https://pathways.embl.de (accessed on 21 December 2023)]; Table S1: Participant characteristics.
