# Supplementary material for: Exposure to Molybdate Results in Metabolic Disorder: An Integrated Study of the Urine Elementome and Serum Metabolome in Mice
Source: Toxics. 2024 Apr 14;12(4):288. doi: 10.3390/toxics12040288 (PMC11053804; doi:10.3390/toxics12040288)
Supplement: Supplementary file 1 [file toxics-12-00288-s001.zip › toxics-2825118-supplementary.pdf]

Supplemental materials

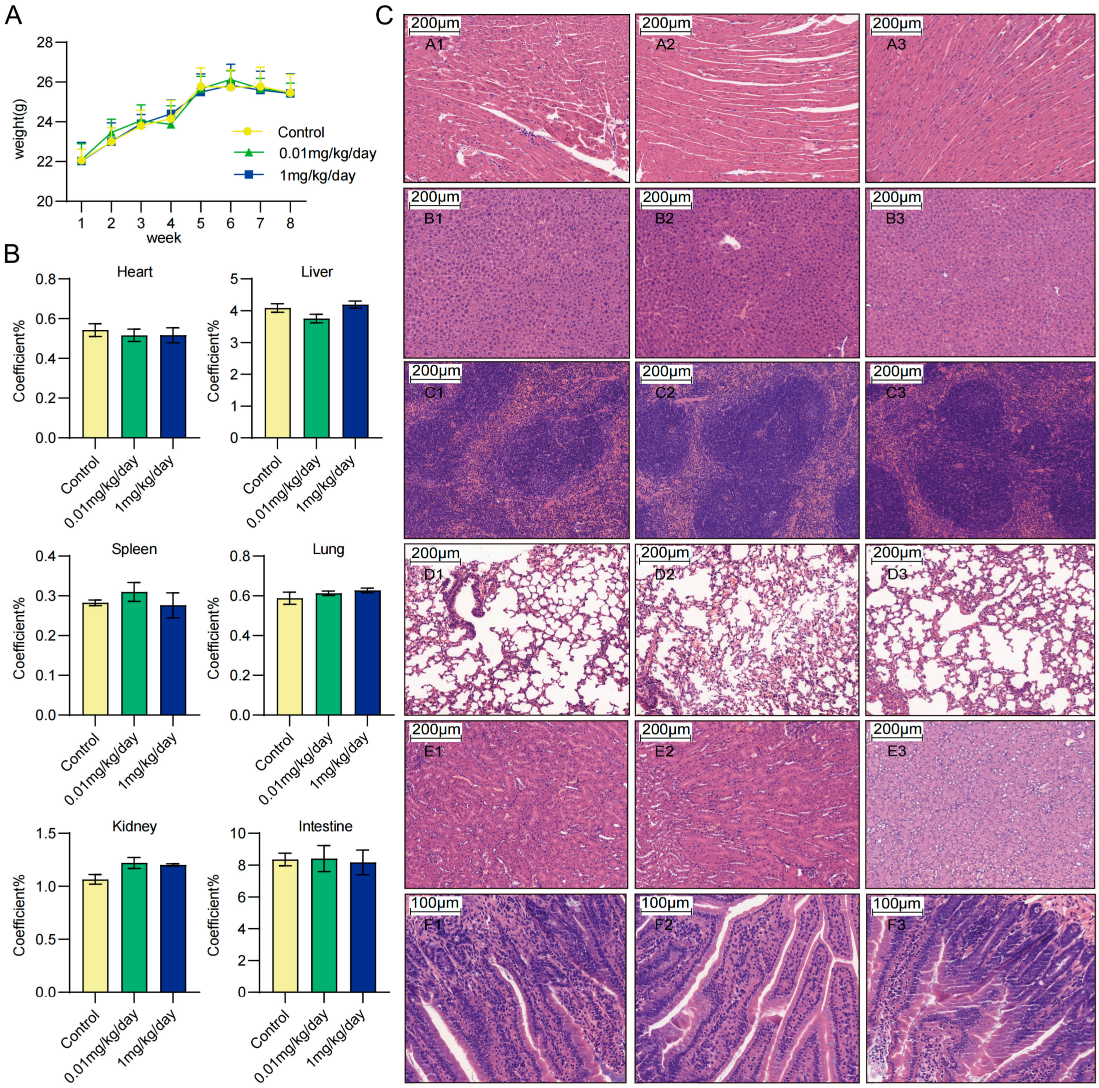

Figure S1. General toxicity of molybdenum exposure *in vivo*. (**A**) Line chart depicting the changes in body weight during molybdenum treatment. (**B**) Histogram illustrating the organ coefficients of the heart, liver, spleen, lung, kidney, and intestine. (**C**) Representative pathological images of the major organs. Figures labeled A to F represent the heart, liver, spleen, lung, kidney, and intestine, respectively. Figures labeled 1 to 3 represent the control group, molybdenum-0.01 mg/kg/day group, and molybdenum-1 mg/kg/day group, respectively. Organ coefficient = weight of the organ (g)/total body weight (g) ×100.

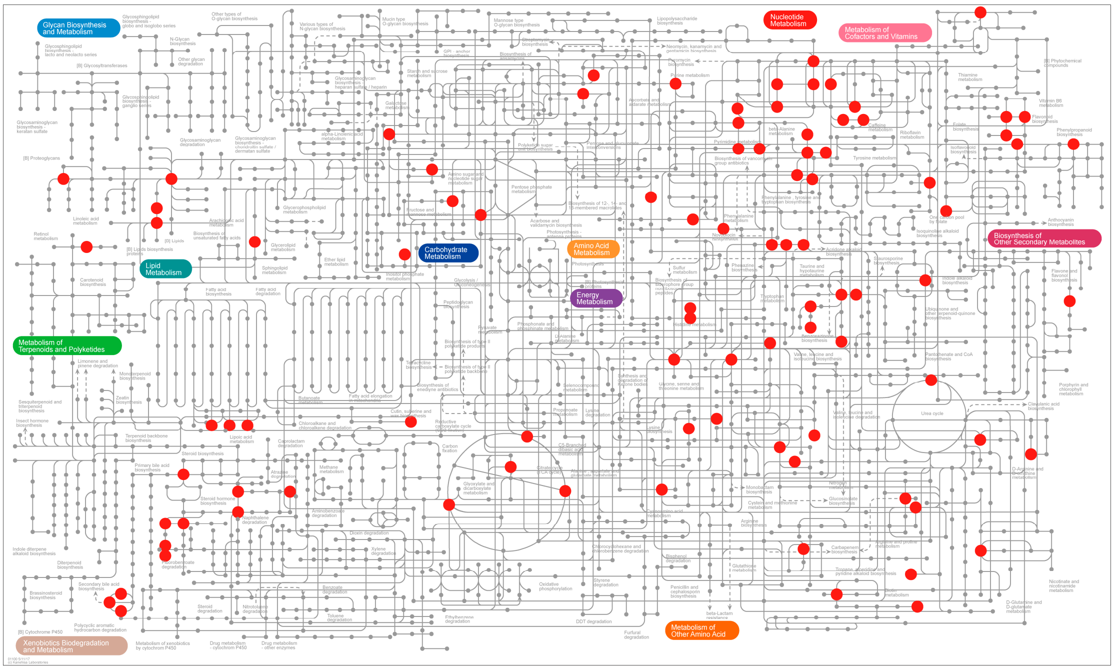

Figure S2. Detected metabolites in the general metabolic pathway based on iPath 3.0 [https://pathways.embl.de (accessed on 21 December 2023)]. The red dot indicates the detected metabolites in serum. The pathway and metabolite information can be found in detail in Metabolism module of Available maps [https://pathways.embl.de/ipath3.cgi?map=metabolic (accessed on 21 December 2023)].

Table S1. Participant characteristics.

		Total Subjects<br>N (%) or Mean (SD)
N		838
Age (Years)		52.14 (17.81)
Race		
	Mexican American	116 (13.8)
	Other Hispanic	70 (8.4)
	Non-Hispanic White	288 (34.4)
	Non-Hispanic Black	200 (23.9)
	Non-Hispanic Asian	113 (13.5)
	Other Race - Including Multi-Racial	51 (6.0)
Education		
	High school and below	185 (22.1)
	High school graduate/GED or equivalent	219 (26.1)
	Some college or AA degree	235 (28.0)
	College graduate or above	198 (23.6)
	Missing	1 (0.1)
Smoking status		
	Never smoker	396 (47.3)
	Former smoker	270 (32.2)
	Current smoker	172 (20.5)
BMI (kg/m^2)		
	Underweight	12 (1.4)
	Normal weight	194 (23.2)
	Overweight	297 (35.4)
	Obesity	320 (38.2)
	Missing	15 (1.8)
Cadmium (μg/L)		0.34 (0.46)
Molybdenum (μg/L)		53.40 (51.70)

BMI, body mass index.

AA degree, an associate's degree.
